# Case of Sarcocele, in Which, after the Removal of One Testicle, the Disease Attacked the Other, and Was Cured by the Use of Bougies

**Published:** 1829-10

**Authors:** John North

**Affiliations:** Surgeon


### SARCOCELE.

*Case of Sarcocele, in which, after the removal of one Testicle, the Disease attacked the other, and was cured by the use of Bougies.* By John North, Surgeon, F.L.S.

In many cases it may be difficult to determine whether the removal of disease is to be attributed to the efficacy of the treatment which is employed, or to some spontaneous operation of nature. I think, however, it may fairly be inferred that the successful termination of the second attack, in the following case, was owing to the means which were adopted.

A gentleman, twenty-six years of age, who had previously enjoyed uninterrupted good health, felt for some weeks a slight tenderness of the left testicle. He attributed it to cold, or fatigue, or some accidental and trifling cause, and paid little or no attention to it. For two months the pain of the testicle continued to increase, but as yet it was not very severe. The part was slightly enlarged. In this state he applied to me. I could not, after the strictest examination, ascertain any probable cause upon which the local affection might be dependent. He had not for many years had gonorrhœa, and had never any symptoms of stricture. He was directed to remain as quiet as possible, and to confine himself to a mild regimen. Leeches were applied to the part, and afterwards fomentations. Occasional purgatives were given, and small doses of calomel, combined with extract of hemlock, twice a day. A large-sized bougie was also once introduced into the bladder, to determine that there was no obstruction in the urethra.

Under this treatment the pain almost entirely ceased for a few days. The testicle, however, continued to enlarge, and it had now an irregular feel. Under the direction of two eminent surgeons, various means, both general and local, were had recourse to; but without avail. The pain and swelling of the part slowly increased for three or four months, and, as the health of the patient was visibly declining from constant suffering, and every mode of treatment had been adopted from which relief could be rationally expected, it was deemed expedient to remove the testicle. It was now at least six times the natural size, and exquisitely painful. The colour of the scrotum was darker than natural. The patient cheerfully consented to the proposal, and the operation was performed.

Upon examining the testicle after its removal, it was found to be completely altered in structure. It presented the appearance of a scrofulous mass. In some parts it was very firm, in others comparatively soft. There was a very small quantity of fluid in the tunica vaginalis.

After the operation the patient speedily rallied, and in the course of a few weeks he was capable of pursuing his accustomed avocations. His health and spirits were completely restored.

About four months from this period he perceived, in walking or riding, a very trifling uneasiness in the right testicle, which would not probably have attracted his attention, had he not been taught by his former experience the necessity of timely precaution. He came to town immediately, in a state of much anxiety. I examined the part attentively, and found that it was tender to the touch, and rather harder than natural. It was nearly double the ordinary size; but it was at first doubtful whether this circumstance was to be attributed to disease, as, after the removal of one testicle, the other frequently becomes much enlarged. Mr. Heaviside, who had before seen the patient, was again consulted.

It would be useless to detail minutely the different local and general remedies that were employed. It was evident that the disease was gradually gaining ground, and that it was pursuing the same course as the former. A slight and very temporary amendment, indeed, was perceived while he was under the influence of a mild course of mercury; at which time, hemlock poultices were also applied to the part.

Mr. Guthrie's opinion was now taken. The testicle was about four times the natural size, and very painful on the slightest pressure; the general health of the patient bad; his mind, of course, anxious and dispirited. Mr. Guthrie recommended the continuance of decoction of sarsaparilla and the compound calomel pill, which he had been before taking; in addition to which, he directed that a large-sized metallic bougie should be passed three times a week, and kept in the urethra for several minutes.

Considerable irritation was produced by this treatment along the whole course of the urethra, and a slight scalding in making water. For the first few times a trifling hemorrhage also followed the use of the bougie. In the course of a fortnight the size of the testicle was evidently diminished, and there was much less tenderness on pressure. The use of the bougie was continued for nearly three months, and during this time the swelling of the testicle, the pain, and indeed every appearance of disease, gradually subsided. The patient could take active exercise without the slightest uneasiness.

Several years have now elapsed since the occurrence of the disease. The gentleman has enjoyed perfect health, and is in full possession of the power of which he was once so much in danger of being altogether deprived; for there was every reason to fear that the remaining testicle must be removed.

It was the opinion of the late Mr. Ramsden, that some cases of sarcocele might be relieved by removing, with bougies, a morbid irritability of the urethra, which he presumed, theoretically, to be sometimes connected with the origin of the complaint. Whether the case I have related tends to support this doctrine, I will not venture to determine. The efficacy of the practice to which it leads cannot, I think, in this instance, be doubted.

Mr. Cooper,* (in commenting upon the use of bougies in cases of sarcocele, as recommended by Mr. Ramsden,) remarks, " the novelty of this suggestion for a time attracted considerable notice; but the interest which it once excited has now died away; a sufficient proof to my mind that the practice inculcated was not of much value." Unless I am mistaken, Mr. Cooper much underrates the confidence which the most competent surgical authorities still place in the treatment suggested by Mr. Ramsden, although it may not, perhaps, be in such general use as to render an additional proof of its *occasional* efficacy altogether superfluous.

It is remarkable that the brother of the gentleman whose case I have above related was afterwards attacked with a similar disease, in consequence of which it became necessary to remove both the testicles. He died of pulmonary consumption. I am unacquainted with the progress of the disease, or with the treatment that was adopted in this case.

*Upper Berkeley street, Portman square.*

* Surgical Dictionary, fifth Edition, p. 1051.

